# Measuring the shape of mortality across animals and plants: Alternatives to *H* entropy metrics reveal hidden type IV survivorship curves and associations with parental care at macro‐ecological scales

**DOI:** 10.1002/ece3.10076

**Published:** 2023-05-17

**Authors:** Kevin Healy, Ruth Kelly, Angela Carnevale, Yvonne M. Buckley

**Affiliations:** ^1^ School of Natural Sciences, Ollscoil na Gaillimhe University of Galway Galway Ireland; ^2^ School of Natural Sciences, Zoology Trinity College Dublin Dublin Ireland; ^3^ Environment and Marine Sciences Division Agri‐Food and Biosciences Institute Belfast UK; ^4^ School of Mathematical and Statistical Sciences, Ollscoil na Gaillimhe University of Galway Galway Ireland

**Keywords:** demography, H‐entropy, life history, mortality, survivorship

## Abstract

The shape of mortality, or how mortality is spread across an organism's life course, is fundamental to a range of biological processes, with attempts to quantify it rooted in ecology, evolution, and demography. One approach to quantify the distribution of mortality over an organism's life is the use of entropy metrics whose values are interpreted within the classical framework of survivorship curves ranging from type I distributions, with mortality concentrated in late life stages, to type III survivorship curves associated with high early stage mortality. However, entropy metrics were originally developed using restricted taxonomic groups and the behavior of entropy metrics over larger scales of variation may make them unsuitable for wider‐ranging contemporary comparative studies. Here, we revisit the classic survivorship framework and, using a combination of simulations and comparative analysis of demography data spanning the animal and plant kingdoms, we show that commonly used entropy metrics cannot distinguish between the most extreme survivorship curves, which in turn can mask important macroecological patterns. We show how using *H* entropy masks a macroecological pattern of how parental care is associated with type I and type II species and for macroecological studies recommend the use of metrics, such as measures of area under the curve. Using frameworks and metrics that capture the full range of variation of survivorship curves will aid in our understanding of the links between the shape of mortality, population dynamics, and life history traits.

## Graphical abstract

We revisit the classic framework of survivorship curves and test whether commonly used entropy measures of these curves are appropriate for contemporary comparative analysis. We show that entropy measures are not appropriate for wide‐scale taxonomic analysis and that entropy measures may mask important macroecological patterns.

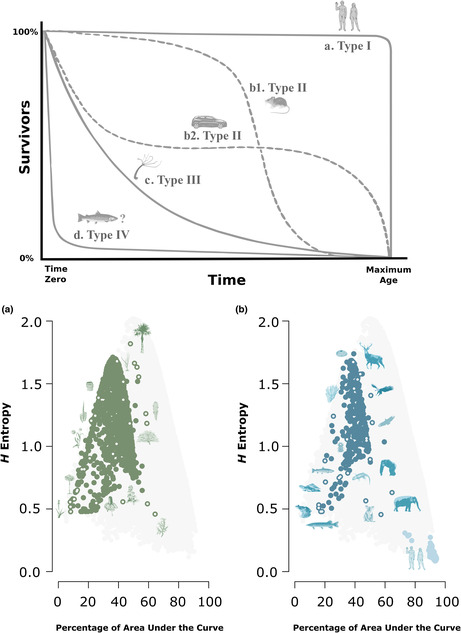

## 1 INTRODUCTION

Mortality is inevitable for all living organisms. However, the timing of mortality for individuals can be markedly different within populations and between species. For example, in many plant species, most individuals will face mortality early in the life course (Salguero‐Gómez et al., 2015), whereas, in contrast, individuals in species such as orcas (*Orcinus orca*) and humans (*Homo sapiens*) are more likely to face death at stages much closer to their maximum ages (Caswell, 2000; Jones et al., 2014; Salguero‐Gómez, Jones, Blomberg, et al., 2016). This spread of mortality across the life cycle is a fundamental component of life history. An organism with extremely high mortality in early life stages faces different evolutionary selection pressures compared with species where individuals typically reach maturity and later life stages (Roper et al., 2021; Stearns, 2000). In turn, these selection pressures are linked with various functional traits associated with reproduction (Price, 1974), ecological strategies (Healy et al., 2019; Kelly et al., 2021), and senescence (Jones et al., 2014; Roper et al., 2021). Within applied fields differences in the timing of mortality have important consequences for the ability of populations to respond to environmental changes and consequently for how we design conservation and management policies (Halley et al., 2018). From a human perspective, variation in the distribution of mortality can be an important indicator of social inequality in health and other socioeconomic factors (van Raalte et al., 2018). Given the fundamental nature of the distribution of mortality, understanding its variation across the tree of life is likely to provide insights into ecological, evolutionary, and population dynamics. However, understanding this variation at the widest taxonomic scales requires comparative frameworks that effectively capture the full range of variation in schedules of mortality between populations and species.

**FIGURE 1:**
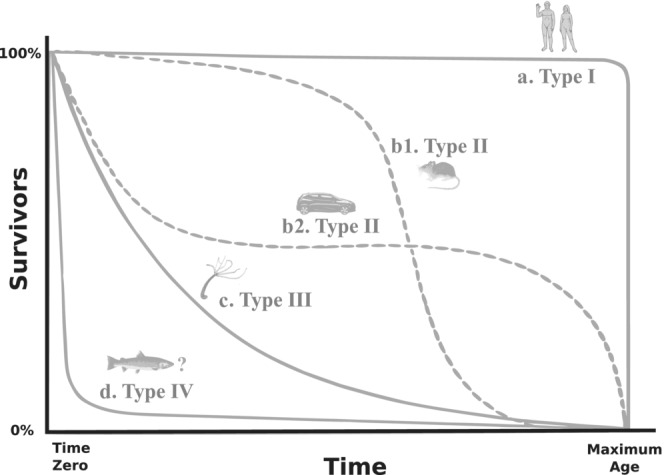
Diagram of theoretical survivorship curves based on Figure 2 from Pearl and Miner (1935). Curves are standardized to end at the maximum age of a given organism with each curve representing how mortality is distributed across the life course. These curves follow the classic type I (a) which is typically represented by humans, type II (b1, b2) with more evenly spread mortality as represented by the mouse and automobile, and type III (c) which has higher early mortality and which was originally represented by Hydra. As described in Pearl and Miner (1935), there is also the theoretical type IV which is likely to be represented by species such as fish which have extremely high early stage mortality rates followed by greatly reduced adult mortality rates.

One of the most widely used frameworks to compare the spread of mortality across populations and species was the use of survivorship curves first outlined by Pearl and Miner (1935). They defined a framework describing how population survivorship declines over the life course of the population according to three main curves: type I survivorship curves describe populations, where mortality is concentrated toward the maximum ages of individuals; type II describes populations where mortality is spread more equally; and type III describes populations with high early stage mortality (Pearl & Miner, 1935). Due to the lack of demographic data this framework initially only described a handful of species, with the authors even using the mechanical decay of motor cars as an example of a type II survivorship curve (Figure 1). Furthermore, Pearl and Miner had outlined an additional type IV survivorship curve to describe species with extreme early stage mortality, noting that while data describing such species was not available “An example would presumably be pelagic breeding types of teleost fishes, where enormous numbers of eggs and larval forms are produced, of which only relatively few survive, but those that do survive have a long life span” (Pearl & Miner, 1935). Although type IV survivorship curves have since dropped out of the lexicon of the field, analyses which include such species are now commonplace (Baudisch et al., 2013; Capdevila et al., 2020; Healy et al., 2019; Jones et al., 2014; Kelly et al., 2021; Roper et al., 2021; Salguero‐Gómez, 2018; Salguero‐Gómez, Jones, Blomberg, et al., 2016). However, while the survivorship curve framework is a useful guide in describing the qualitative distribution of mortality across life histories, comparative analyses require quantitative metrics of mortality spread which can subsequently be reliably assigned to survivorship curve types from type I to type IV.

Numerous measures have been developed that aim to quantify the shape of mortality. These include metrics that rely on various aspects of survival including coefficients of variation, hazard functions, Gini index and using ratios of mortality, area under the curve (AUC) measures from survivorship curves, and measures using survival from different time points of the life course, e.g., *H*‐ratio (Ebeling et al., 2018; Wrycza et al., 2015). One of the earliest of these approaches that are frequently used in comparative analysis (Beckman et al., 2018; Capdevila et al., 2020; Connor et al., 2020; Salguero‐Gómez, Jones, Blomberg, et al., 2016), is the use of entropy measures as outlined by Demetrius (1974, 1979), and further developed by Keyfitz (1977).

Entropy measures are related to the wider concept of information theory and in demography, they are applied to measure the information contained in survivorship curves (*lx*) to define the effects of some proportional change in mortality (Keyfitz, 1977). These entropy measures, often referred to as *H* or Keyfitz entropy, can also measure the convexity of the curve, with derivations typically having values of *H* close to 0 for type I, *H* = ½ for type II and increasing values of *H* expected to be associated with type III (Goldman & Lord, 1986). Although *H* entropy is well defined over the range of type I and II survivorship curves and has typically been applied to species within this narrow range in comparative studies (Capdevila et al., 2020; Halley et al., 2018; van Raalte et al., 2018), the behavior of these metrics for type III and potentially more extreme type IV survivorship curves is less clear. In particular, as *H* entropy is fundamentally a measure of information extreme type IV curves may give similar values to those of the most extreme type I survivorship curves. This can arise as populations with extremely high initial mortality rates may also display such low adult mortality rates that they follow the trajectory of a type I curve for the majority of their life history course. Furthermore, the issue of similar entropy values being assigned to clearly different survivorship curves can be demonstrated mathematically by splitting the term describing *H* entropy into two parts (See Appendix S1). However, while this behavior has the potential to cause significant issues in the interpretation of comparative analyses of mortality distribution, whether problematic values of *H* entropy arise in observed survivorship data have yet to be tested or explored.

The availability of demographic data for species representing groups across the animal and plant Kingdoms has greatly expanded over the last couple of decades. With the advent of open databases such as the COMADRE animal matrix database (Salguero‐Gómez, Jones, Archer, et al., 2016) and COMPADRE plant matrix database (Salguero‐Gómez et al., 2015) along with comparative tools (Jones et al., 2022) and widely available phylogenies (Hinchliff et al., 2015), comparative analysis of life history strategies and demographic patterns are now possible at the scale of Kingdoms (Healy et al., 2019; Jones et al., 2014; Kelly et al., 2021; Salguero‐Gómez, Jones, Blomberg, et al., 2016). With this explosion of new data demographers and ecologists no longer face the taxonomic limitations of early analyses and can now fully explore the extent of variation in the shape of mortality across life forms and phylogenies.

Capturing variation in the spread of mortality can have important consequences for how we understand the relationships between life history parameters, functional traits, and the niche an organism occupies. For example, large‐scale comparative analyses have found links between life history strategies and a species ecological mode of life (Healy et al., 2019), habitat type (Capdevila et al., 2020), and climate (Kelly et al., 2021). However, there have been few specific links made between life history and the shape of mortality, despite clear expectations. For example, investment into offspring is expected to be directly linked with juvenile mortality, with life history strategies that involve high parental investment into fewer offspring expected to be associated with mortality distributions trending toward type I survivorship curves (Clutton‐Brock, 2019). Such variation in offspring investment has been found to be an important component in life history strategies independent of the classic fast‐slow continuum (Stearns, 1989), however, whether it is directly linked to the spread of mortality has yet to be tested.

Here we use a combination of empirical demographic data and simulations to explore the full range of variation in the shape of mortality in plants and animals. Within this range of mortality distributions, we then compare the ability of *H* entropy against AUC metrics to ascertain whether *H* entropy can capture the range of survivorship variation in observed populations, from type I populations, such as humans, to potential type IV species, such as grasses, fish, and corals. Finally, we use the example of the relationship between parental care and the spread of mortality to show that, as expected, the presence of parental care is more associated with species with type I and type II survivorship curves when using metrics such as AUC. This example also shows how *H* entropy can miss fundamental patterns and highlights the importance of metric choice when analyzing macroecological patterns of the spread of mortality.

## 2 METHODS

To investigate the behavior of *H* entropy across the full span of possible and realized survivorship curves, we use a combination of simulated and empirical survivorship curves using available demographic data in the form of population matrix models for animals and plants. We further test the importance of metric choice by testing the relationship between survivorship curve type and the reproductive output of animals.

### 2.1 Simulated data

To simulate distributions of age‐specific mortality for a wide range of potential survivorship curves, including those not observed in nature, we simulated both random curves and curves generated from the Gompertz equation (Gompertz, 1825). For randomly generated curves, we created 1,000,000 population matrix models of dimension 10 with randomly assigned age‐specific mortality rates, between 0 and 1, for each stage. From these simulated matrices, we calculated age‐specific survivorship curves (*l*_*x*_) using age‐from‐stage decompositions outlined by Caswell (2000). We ended the sequence for all *l*_*x*_ curves once survivorship reached below <99.9% of the initial population size. To ensure we captured the full range of biologically realistic curves, we also generated 1,000,000 curves with simulated juvenile and mortality rates. Values for juvenile mortality rates were randomly drawn from a uniform distribution between 0.99 and 0.01 and applied for a random number of initial time steps between 1 and 4 to represent the juvenile age states. For each simulation, an adult mortality rate was then randomly drawn from a uniform distribution between 0.1 and 0.01 and set to monotonically decline until *l*_*x*_ = 0.

While using randomly generated population matrices should theoretically provide the full range of possible monotonically declining survivorship curves, the probability of generating a type I curve using this method can be low. Hence, to ensure we captured the full range of possible curves we also simulated survivorship curves using the Gompertz equation which is ideal for generating patterns associated with type I curves (Sas et al., 2012). Curves simulated based on Gompertz law were simulated using the equation.

(1) $$S_{t}=S_{0}e^{c\left(1-e^{\mathrm{kt}}\right)},$$

where $S_{t}$ is the proportion of a population surviving at time *t* for an initial population of $S_{0}$, here set to 1 so all values are proportional, and both *c* and *k* are population‐specific parameters (Sas et al., 2012) which define the shape of the curve. We generate 500,000 curves by drawing with replacement values between 0 and 20 for *c* and between 0 and 1 for *k* according to a uniform distribution. To ensure we simulated type I curves using this approach we also simulated an additional 500,000 curves with values of *c* selected from a uniform distribution between 0 and 0.1.

### 2.2 Empirical data

For observed populations, we used matrix population models from the COMADRE Animal Matrix Database vr 3.0.1 (Salguero‐Gómez, Jones, Archer, et al., 2016), the COMPADRE Plant Matrix Database vr 4.0.1 (Salguero‐Gómez et al., 2015) and life tables from Keyftz & Flieger (Flieger, 1990; Keyfitz & Flieger, 1968, 1971) for human populations. The COMADRE and COMPADRE databases contain demographic data compiled as age, size‐ or developmental stage‐structured matrix population models (MPMs). We used the mean and pooled MPMs from the COMADRE and COMPADRE databases, which are calculated as element‐by‐element arithmetic means of matrices across all study periods and sites under the same treatment conditions for a species within a study (Salguero‐Gómez et al., 2015; Salguero‐Gómez, Jones, Archer, et al., 2016). We only included MPMs that were parameterized from noncaptive populations in un‐manipulated conditions, and which could be divided into separate sexual and clonal reproduction matrices. For plants, when seed‐stage data were available, this was included in life history calculations to incorporate the full range of life history spread. To ensure that each MPM represented a complete life cycle, we only included those that were irreducible, primitive, and hence ergodic, as tested using the popdemo package (Stott et al., 2012). Irreducible matrices have a direct or indirect pathway between all stages while primitive matrices do not exhibit long‐term cyclical population dynamics (Stott et al., 2010). For each MPM, we calculated the age‐specific survivorship curve (*l*_*x*_) using age‐from‐stage decompositions outlined by Caswell (2000). Our final data set included 305 plant species represented by 905 populations and 116 animal species represented by 278 populations.

### 2.3 Measuring the shape of mortality

For each simulated and observed survivorship curve *l*_*x*_, we calculated *H* entropy (*H*) using the kentropy function in the Rage package (Jones et al., 2022) which follows Demetrius's (1978) formulation:

(2) $$H=-\frac{\int_{0}^{\infty }l\left(x\right)\log l\left(x\right)\;\mathrm{dx}}{\int_{0}^{\infty }l\left(x\right)\;\mathrm{dx}}.$$

As the length of the sequence for an *l*_*x*_ curve may affect *H* entropy values, as longer sequences may be able to show more extreme values at each end of the range, we also calculated *H* entropy by first fitting splines to each *l*_*x*_ curve and using 50 evenly spaced inferred values across the sequence to estimate *H* entropy according to equation (2). To compare *H* entropy values to other measures of the shape of mortality, we used the area under the *l*_*x*_ curve (AUC) as a proportion of the rectangle defined by all individuals surviving to the last step in the *l*_*x*_ curve (i.e. the most extreme theoretical type I curve). AUC values close to 1 indicate type I curves, with declining values indicating type II to type III and IV curves for values close to 0. We calculated AUC both using the above non‐standardized approach and also using the standardized approach from the Rage package which standardizes the AUC against a constant survival function which results in giving values between −0.5 and 0.5 (Jones et al., 2022).

### 2.4 Relating the shape of mortality to parental investment

As large‐scale comparative analyses typically aim to test fundamental evolutionary patterns and predictions, we explore how extending the analysis of life history data to include type IV survivorship curves influences expected fundamental patterns associated with parental investment. Due to the extremely high juvenile mortality rates associated with type IV or III curves, species with these life histories are not expected to invest in high levels of parental care. In contrast, species where mortality is more concentrated in later life stages, and where investment into juveniles is less likely to be lost are expected to be associated with increased parental care. To test the potential relationship between curve type and reproductive output, we use the demography data and phylogeny from Healy et al. (2019) that represents 116 animals. For these species, we collated data on the presence of parental care, from various sources (del Hoyo et al., 1992; Froese & Pauly, 2010; Mittermeier & Wilson, 2009), which we defined as continued protection or feeding postbearing offspring. To test our hypotheses, we fitted Bayesian phylogenetic mixed models (BPMM) using the MCMCglmm package (Hadfield et al., 2019) in R version 4.1.0 (Team, 2021). We controlled for pseudoreplication due to shared ancestry between species by using the animal term in MCMCglmm (Hadfield, 2010). The animal term uses a distance matrix of the phylogenetic distance between species to control for the expected similarity in trait values due to phylogenetic relatedness. We calculated the term h^2^ as the relative variance attributable to the animal term (Hadfield & Nakagawa, 2010). This term can be interpreted in a similar fashion to the phylogenetic lambda value, with a *h* ^2^ value close to 1 indicating a Brownian model of trait evolution, and a value close to 0 indicating independence between trait values (Hadfield & Nakagawa, 2010). We fitted all models using parameter‐expanded priors, with standard noninformative priors also tested separately to ensure that the choice of prior had no effect on model results (Hadfield, 2010). A burn‐in of 100,000, thinning of 500, and 1,100,000 iterations were used as they resulted in an effective sample size that exceeded 1000 for all parameter estimates. We tested for convergence using the Gelman‐Rubin statistic over three separate chains (Brooks & Gelman, 1998). To incorporate the error associated with building phylogenies, we used the mulTree package (Guillerme & Healy, 2014) and the phylogeny constructed in (Healy et al., 2019) which consists of a distribution of 100 supertrees constructed using available phylogenies (Betancur‐R et al., 2013; Bininda‐Emonds et al., 2007; Jetz et al., 2012; Pyron & Burbrink, 2014), with the open tree of life (Hinchliff et al., 2015) used as a backbone (See Healy et al., 2019 for further details). We used mulTree to run each separate MCMCglmm model for each of the 100 trees, with the posterior distributions then combined. We ran a separate such analysis for *H* entropy and AUC with the presence absence of parental care as the explanatory variable.

## 3 RESULTS

### 3.1 Simulated data

**FIGURE 2:**
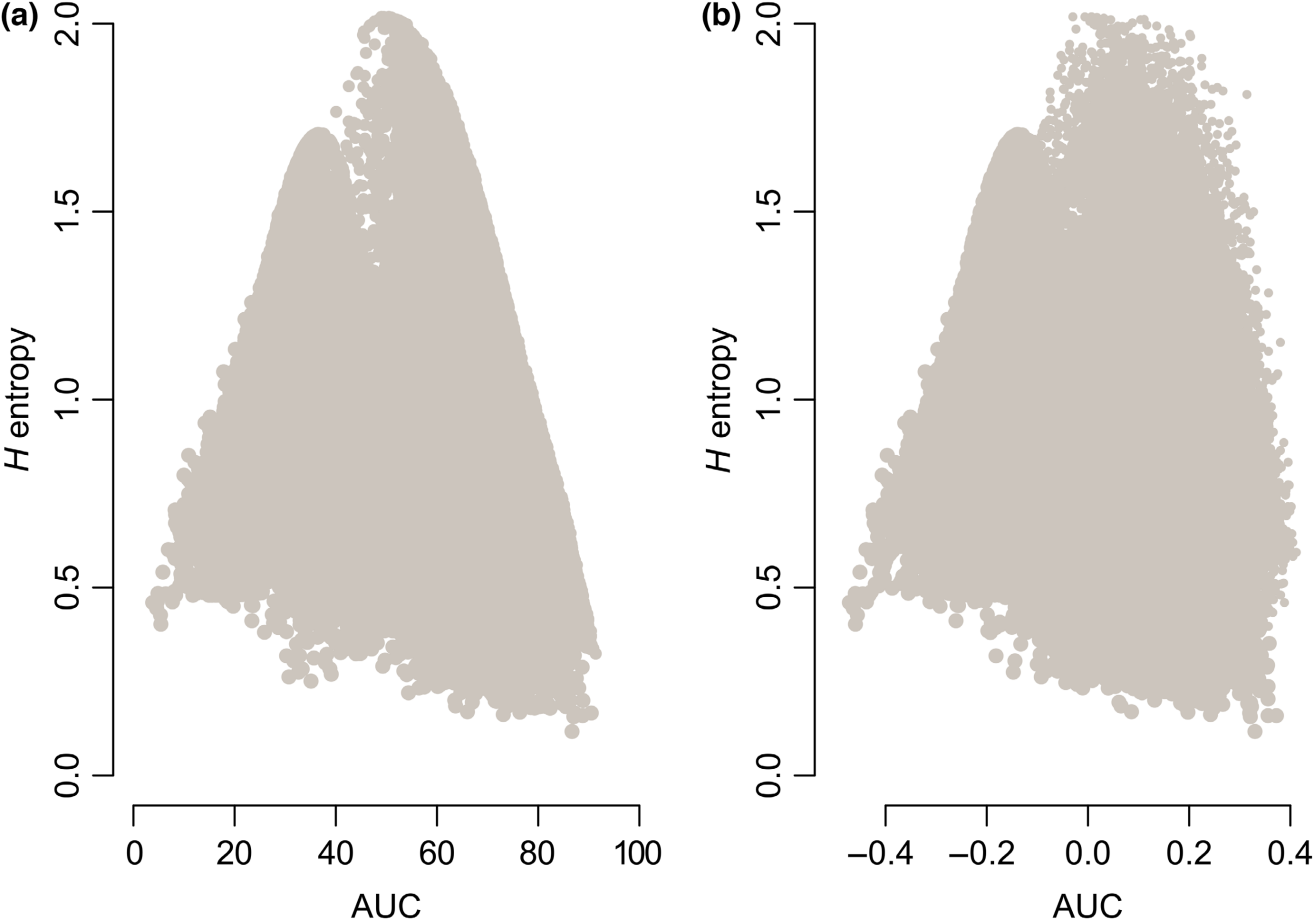
Relationship between *H* entropy, (a) corrected for length of *lx* series and AUC, (b) AUC measured using the Rage package for 3,000,000 simulated survivorship curves. Each graph demonstrates how *H* entropy has a concave relationship with AUC (a, b) and in extension with survivorship curve type with low *H* entropy values representing both type I and type IV survivorship curves. See Figure S2 for a breakdown of each simulation type.

We generated simulated data for survivorships curves covering the theoretical range between the values of 0.1–2.0 when calculated using *H* entropy, between 0.04 and 0.91 for AUC and between −0.47 and 0.41 for AUC calculated using Rage, which is standardized using a constant survival function (Figure 2). When *H* entropy is compared with AUC (Figure 2a), we find a clear humped relationship where the highest *H* entropy values, those greater than 1.5, corresponding to intermediate values of AUC between the range of 0.3 and 0.7, whereas the lowest *H* entropy values are associated with both the highest and the lowest AUC values (Figure 2). Hence, although the lowest AUC values represent type IV survivorship curves and the highest AUC values represent type I survivorship curves, enabling clear mapping between AUC and survivorship curve type, we find that the lowest *H* entropy values represent both type IV and type I survivorship curves (Figure 2a). This pattern is also found when comparing *H* entropy against both the AUC as calculated in the rage package (Figure 2b). These concave relationships are also found when using *H* entropy measures not corrected for the length of the *lx* series (Figure S1).

### 3.2 Empirical data

**FIGURE 3:**
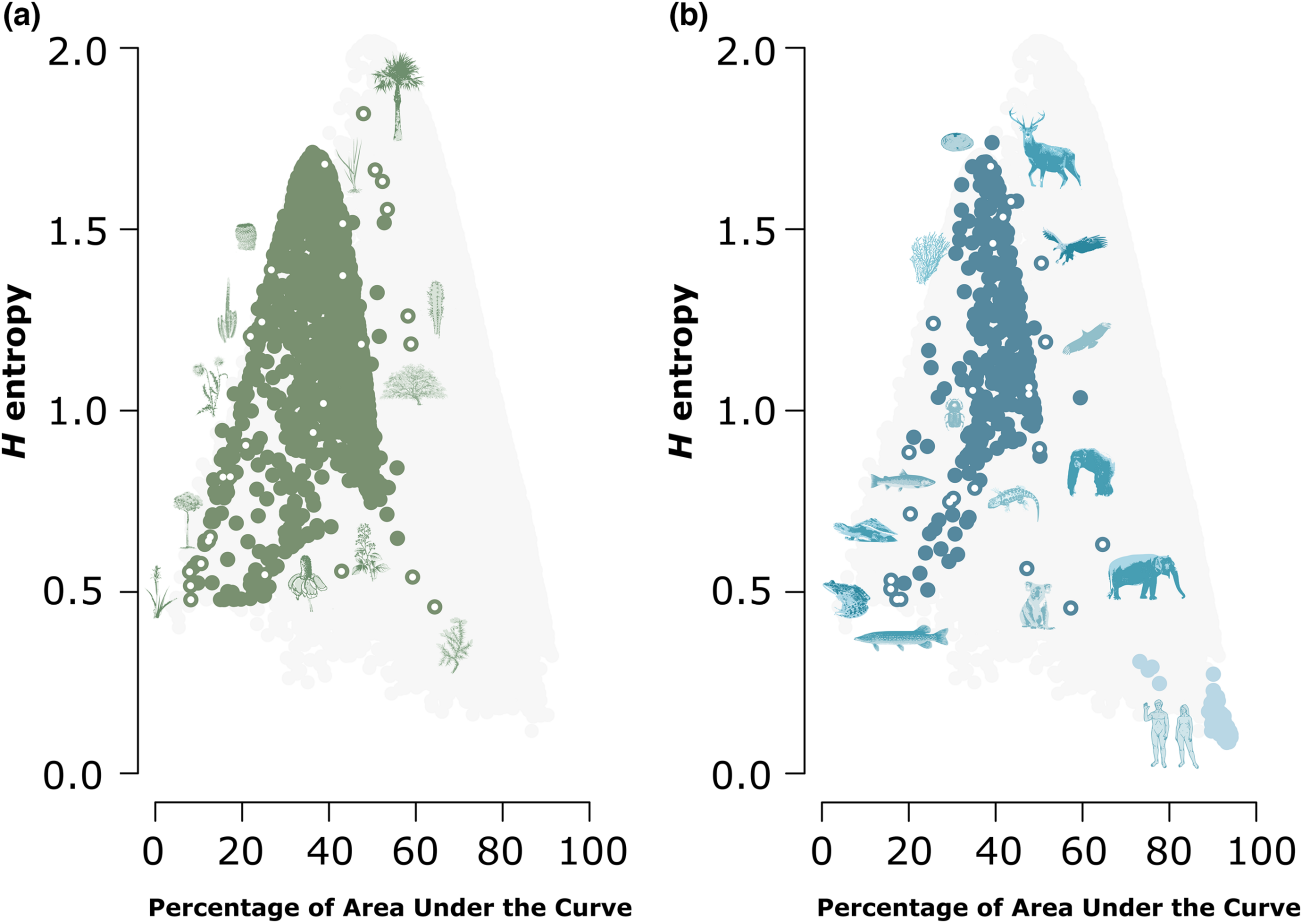
Relationship between *H* entropy, corrected for length of *lx* sequence, and the area under the *lx* curve as a percentage of the highest possible area represented by a square for the most extreme type I curve (AUC). For both plants (a, green, 905 populations representing 305 species) and animals (b, blue, 278 populations representing 116 species), the relationship follows a clear concave relationship with both type I and type IV curves represented by low *H* entropy values, whereas type II is represented by the highest *H* entropy values. The range of simulated data is indicated by the gray silhouette in the background. Species highlighted with light‐colored canters from left to right in (a) *Dactylorhiza lapponica*, *Vochysia ferruginea*, *Carduus nutans*, *Neobuxbaumia macrocephala*, *Mammillaria magnimamma*, *Ratibida columnifera*, *Rubus praecox*, *Molinia caerulea*, *Thrinax radiata*, *Escontria chiotilla*, *Prosopis glandulosa*, *Ulex gallii*, and from left to right and in (b) *Rana luteiventris*, *Esox Lucius*, *Emydura macquarii*, *Salvelinus confluentus; Paramuricea clavata; Scolytus ventralis; Sceloporus grammicus*, *Mya arenaria*, *Cervus elaphus*, *Haliaeetus albicilla*, *Coragyps atratus*, *Pan troglodytes*, *Phascolarctos cinereus*, *Elephas maximus*, *Homo Sapiens (light blue)*.

While we found a concave relationship between *H* entropy and survivorship curve type in our simulated data, we tested whether such a relationship is found in realized survivorship curves observed in animal and plant populations. For animals, *H* entropy values ranged from 0.09 in humans (*Homo sapiens*) to 1.74 in *Crocodylus johnsoni*. The most extreme type I survivorship curve, as represented by AUC values, was 0.94 in humans (*Homo Sapiens*) with the most extreme type IV found in the Columbia spotted frog (*Rana luteiventris*) with an AUC of 0.16 (Figure 3b). In plants, *H* entropy values ranged from 0.46 in western gorse (*Ulex gallii*) to 1.8 in Florida thatch palm (*Thrinax radiata*). Based on AUC, the Lapland marsh‐orchid (*Dactylorhiza lapponica*) represented the most extreme type IV survivorship curve (AUC 0.08), whereas the western gorse (*Ulex gallii*) represented the most extreme type I (AUC 0.64; Figure 3a).

For both plants and animals, we find a clear concave pattern between *H* entropy and survivorship curve with low *H* entropy values representing both type I and type IV populations. This agrees with our simulation results and indicates that *H* entropy also does not accurately characterize these survivorship curve types in observed plant and animal populations. While the observed survivorship curves do not represent all possible theoretical curves in the simulated data set, they are observed across almost the full range of simulated curves. Interestingly, the rarest survivorship curves, based on available data are not type IV or type III but type I survivorship curves, such as those found in humans (Figure 3).

### 3.3 Relating the shape of mortality to reproductive investments

**FIGURE 4:**
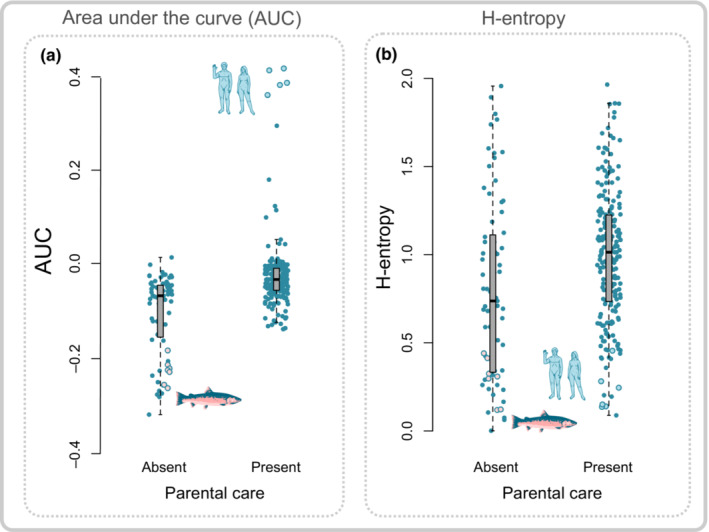
Boxplots for AUC, as measured using the Rage package, and *H* entropy values for species with parental care (Present) compared with those without parental care (absent). (a) Parental care is significantly associated with higher AUC values indicative of type I survivorship curves (β = 0.08, lower 95% CI = 0.01, higher 95% CI = 0.01). In contrast, (b) we do not find a significant relationship between parental care and *H* entropy. Issues regarding *H* entropy's ability to distinguish between type I and type IV species are highlighted by how Humans (*Homo sapiens*) and Chinook salmon (*Oncorhynchus tshawytscha*) are clearly distinguished using AUC but are given similar values when using *H* entropy. *N* = 278 populations representing 116 species, 83 with parental care and 33 without.

To test how including the full range of survivorship types may affect the ability of comparative analysis to identify fundamental macroecological patterns, we tested whether parental care was more associated with type I survivorship curves using *H* entropy and AUC measures. We found that both AUC measures show that species with more type I survivorship curves are associated significantly more with parental care (Table 1, Tables S1 and S2, Figure 4). In contrast, when using *H* entropy, a significant effect between survivorship curve type and the presence of parental care is not found (β = 0.27, lower 95% CI = −0.24, upper 95% CI = 0.80). This nonsignificance is likely due to the lack of differentiation between type IV and type I survivorship curves when measured using *H* entropy as demonstrated by similar values for a typical type I species (humans, *Homo sapiens*) and a type IV species (Chinook salmon, *Oncorhynchus tshawytscha*) when measured using *H* entropy (Figure 4b).

**TABLE 1:** Results of models testing whether the shape of mortality in species with parental care is skewed toward type I survivorship curves.

AUC model	Estimate (β)	Lower 95% CI	Higher 95% CI
Fixed terms			
*Intercept*	−0.08	−0.16	0.03
*Parental care present*	0.08	0.01	0.14
Random terms			
*Residual*	0.13	0.06	0.20
*Species*	0.27	0.12	0.55
*Phylogenetic*	0.60	0.28	0.82

**H‐entropy model**	**Estimate (β)**	**Lower 95% CI**	**Higher 95% CI**
Fixed terms			
*Intercept*	1.00	0.12	1.88
*Parental care present*	0.27	−0.24	0.80
Random terms			
*Residual*	0.07	0.04	0.13
*Species*	0.04	0.01	0.15
*Phylogenetic*	0.88	0.73	0.96

*Note*: The AUC model measures the shape of mortality using area under the curve (AUC), whereas the *H* entropy model uses the entropy measure, both using the Rage package in R (see methods). The estimate (β) is reported as the mode of the posterior distribution with a higher and lower 95% credibility interval (CI). Fixed terms include the intercept and the effect associated with the presence of parental care, with random terms associated with phylogenetics, within species, and residual variation reported as the proportion of each random term.

## 4 DISCUSSION

With the increasing prevalence of cross‐taxonomic macroecological studies in the ecological literature, it is essential that the comparative tools and frameworks used to quantify and explore variation in life history are fit for purpose at the scale of interest. Here, we show that an overlooked aspect of the behavior of entropy metrics undermines its use as a comparative metric across the largest levels of variation of possible survivorship curves. Specifically, we find from both mathematical analysis and simulations that *H* entropy metrics do not differentiate between cases where juvenile mortality is very high (type III/IV survivorship curves), and cases where the majority of mortality occurs in later stages (type I survivorship curves). Moreover, we demonstrate that this issue can arise in comparisons of survivorship curves at macroecological scales, as variation in survivorship curves in both the plant and animal Kingdom span almost across the full range of theoretical survivorship curves. As demonstrated in an example of how parental care is distributed across the animal kingdom, we find that the use of *H* entropy can mask key macroecological patterns compared with other measures of the spread of mortality.

In the inception of the survivorship framework, the existence of a fourth type of survivorship curve was originally included for species with early stage mortality more extreme than what is now described by the “type III” curve (Pearl & Miner, 1935). This “type IV” curve is a natural counterpart to the most extreme “type I” curves in terms of approaching a rectangular shape with the majority of mortality occurring in the earliest juvenile stages followed by remaining individuals typically surviving to the species' maximum lifespan (Figure 1). The current textbook description of survivorship curves typically no longer describes such type IV curves, instead using type III as a category covering all survivorship curves with more extreme juvenile mortality than type II (Krebs, 1985). While such categorizations are in essence subjective, they can shape how we develop quantitative methods and change the interpretation of quantitative patterns. For example, the interpretation of survivorship curves that were originally viewed as extreme cases but are now categorized with less extreme survivorship curves may overlook the importance of such outliers in comparative analysis of life history.

We demonstrate that entropy metrics, such as *H* entropy, cannot distinguish between type I and type IV survivorship curves. The implications of this imperfect assignment of survivorship curve identity go beyond that of a mathematical curiosity given that the range of observed survivorship curves in plants and animals currently used in macroecological studies (Healy et al., 2019; Jones et al., 2014; Kelly et al., 2021; Salguero‐Gómez et al., 2015; Salguero‐Gómez, Jones, Blomberg, et al., 2016), extend across the ranges where entropy measures are problematic. Interestingly, compared with simulated survivorship curves, we find that the observed curves almost cover the entire range of theoretically possible curves, demonstrating the enormous diversity of mortality distributions across both kingdoms. Moreover, curves describing type III and IV were found to be some of the most common in our analysis, with curves that could be described as type I found to be the rarest. This is not unexpected given that high early stage mortality is commonly observed for many species in nature, with only a handful of species demonstrating clear type I survivorship curves (Healy et al., 2019).

Given the relatively common occurrence of type IV survivorship curves, comparative analyses which aim to uncover general ecological or evolutionary patterns associated with life history hence need to take account whether the chosen metric can capture such variation. As an example, we find in an analysis of the distribution of parental care that entropy measures can erroneously place species such as humans (*Homo sapiens*) close to species such as salmon (*Oncorhynchus tshawytscha*) in terms of their associated survivorship types. As expected, when using other measures of the spread of mortality, such as the area under the curve, we found that species with parental care are more associated with type I and type II life history strategies. This finding reflects the initial predictions that parental care should be rarer in species with shorter lifespan and or lower adult survivorship (Stearns, 1976). It also suggests that among the numerous pathways toward the evolution of parental care (Klug & Bonsall, 2010), parental care is likely to only be maintained if it provides higher juvenile survival rates, such as in type I and type II species. These results also show how, when using the appropriate metric, large‐scale macroecological approaches can demonstrate general patterns relating demography to species ecologies and behaviors.

At more restricted taxonomic scales, such as studies of primates (Colchero et al., 2016), entropy measures, such as *H* entropy, are still likely to be appropriate and useful metrics. Moreover, demographers studying variation in survivorship curves across smaller scales of variation may find that *H* entropy is an appealing metric due to its capacity to describe smaller changes in the shape of the survivorships curve compared with some ratio‐based or AUC metrics. It may also be mathematically appropriate due to its first principles links to the concepts of type I, type II, and type III survivorship curves (Demetrius, 1978) as well as its inherent pace‐standardization and dimensionlessness (Wrycza et al., 2015). However, as we demonstrate above, such metrics do not quantify survivorship curves across the full range of observed variation in nature. Therefore, for broad‐scale comparative studies, we recommend the use of metrics that are monotonic across the range of variation, such as the various AUC measures (Ebeling et al., 2018; Wrycza et al., 2015). Furthermore, as *H* entropy demonstrates, the specific purpose a metric is developed for should be strongly considered for comparative analysis. For example, several metrics, such as those developed by Wrycza et al. (2015), are specifically derived to distinguish between different types of senescence and perform best when applied across adult age ranges. Hence, in large‐scale comparative analysis aspects relating not only to the metric but also to the age range in question should be carefully considered, such as whether including the full life course or excluding juvenile mortality is appropriate to the question or metric used. Just as the choice between a sledgehammer and a pin hammer depends on the scale of the job at hand, the choice between metrics of survivorship also depends on scale. Through choosing the correct tools for the job ecologists and demographers can best explore and test patterns across the wealth of demography and life history information now available to us.

## AUTHOR CONTRIBUTIONS

**Kevin Healy:** Conceptualization (equal); data curation (equal); formal analysis (lead); methodology (lead); project administration (equal); software (lead); visualization (equal); writing – original draft (equal); writing – review and editing (equal). **Ruth Kelly:** Conceptualization (equal); data curation (equal); formal analysis (equal); investigation (equal); methodology (equal); software (equal); writing – original draft (equal); writing – review and editing (equal). **Angela Carnevale:** Conceptualization (equal); formal analysis (equal); investigation (equal); methodology (equal); validation (lead); writing – original draft (equal); writing – review and editing (equal). **Yvonne M. Buckley:** Formal analysis (equal); funding acquisition (lead); investigation (equal); methodology (equal); project administration (lead); supervision (lead); writing – original draft (equal); writing – review and editing (equal).

## FUNDING INFORMATION

Yvonne Buckley: Science Foundation Ireland (15/ERCD/2803), Ruth Kelly: Irish Research Council postdoctoral fellowship scheme (GOIPD/2016/324), Kevin Healy: Irish Research Council COALESCE (2021/117).

## CONFLICT OF INTEREST STATEMENT

The authors declare that they have no conflict of interest.

## Supporting information


Appendix S1.

Appendix S2.

## Data Availability

All data files and R code are available at https://github.com/healyke/Measuring‐the‐shape‐of‐mortality‐across‐Animal‐and‐Plants.
