# Supplementary material for: Measuring the shape of mortality across animals and plants: Alternatives to H entropy metrics reveal hidden type IV survivorship curves and associations with parental care at macro‐ecological scales
Source: Ecol Evol. 2023 May 17;13(5):e10076. doi: 10.1002/ece3.10076 (PMC10191775; doi:10.1002/ece3.10076)
Supplement: Supplementary file 1 — Appendix S1. [file ECE3-13-e10076-s002.pdf]

# APPENDIX A
# OBSERVATIONS ON $H$-ENTROPY AND SURVIVORSHIP CURVES

Let $l_x = l(x)$ be a survivorship curve and let $H$ be the following

$$H = -\frac{\int_0^\infty (\ln l_x) l_x \,\mathrm{d}x}{\int_0^\infty l_x \,\mathrm{d}x}.$$

As pointed out in [1, p. 276], from a mathematical point of view, values of $H$ do not bear any particular relationship to life expectancy. The following family of examples is provided there to support this claim: if $l_x = e^{-\lambda x}$, then $H = 1$ for any value of $\lambda$.

Similarly, one can construct a family of curves whose $H$-entropy will attain any positive real number. Indeed, for $k > 0$ let $l_x^k := e^{-x^k}$. Then

$$H_k = -\frac{\int_0^\infty (\ln e^{-x^k}) e^{-x^k} \,\mathrm{d}x}{\int_0^\infty e^{-x^k} \,\mathrm{d}x} = -\frac{\int_0^\infty -x^k e^{-x^k} \,\mathrm{d}x}{\int_0^\infty e^{-x^k} \,\mathrm{d}x} = \frac{1}{k}.$$

This can be seen by integrating the numerator by parts, as

$$\int x^k e^{-x^k} \,\mathrm{d}x = -\frac{1}{k}\left( x e^{-x^k} - \int e^{-x^k} \,\mathrm{d}x \right).$$

**When most of the population dies close to birth.** Rewriting $H$ as a sum of two terms sheds some light on why this invariant to be, say, close to 1 when a large proportion of the population dies at birth, and the remaining individuals survive until attaining some maximum lifespan.

Suppose a proportion $1 - \kappa$ of the population dies at time $\kappa$ and the remaining $\kappa$ survive until time $\omega$, at which everybody dies. (Note: the corresponding survivorship curve is *not* a continuous function.) In that case, the integrals defining $H$ break into sums as follows:

$$H = -\frac{\int_0^\kappa (\ln 1) \,\mathrm{d}x + \int_\kappa^\omega \kappa(\ln \kappa) \,\mathrm{d}x}{\int_0^\kappa 1 \,\mathrm{d}x + \int_\kappa^\omega \kappa \,\mathrm{d}x},$$

which in turn yields

$$H = -\frac{(\omega - \kappa)\ln \kappa}{1 + \omega - \kappa}.$$

The latter quantity can be made large by choosing $\kappa$ small enough (cf. [1, p. 277]).

One can allow for a further variation as follows. Assuming we normalise the total life expectancy, i.e. renormalise the curves so that all individuals will be dead by time 1, one could allow for a proportion $1 - \kappa$ of the population to die by time $a$ (with $0 < a < 1$), while the remaining proportion $\kappa$ dies at time 1. Then $H$ is as follows:

$$H = -\frac{\int_0^a (\ln 1) \,\mathrm{d}x + \int_a^1 \kappa(\ln \kappa) \,\mathrm{d}x}{\int_0^a 1 \,\mathrm{d}x + \int_a^1 \kappa \,\mathrm{d}x},$$

which in turn yields

$$H = -\frac{(1-a)\kappa \ln \kappa}{a + (1-a)\kappa}.$$

One can take $a$ very close to 0 and vary the proportion of survivors $\kappa$ to produce several values of $H$ that get close to or indeed exceed 1.

## References

[1] N. Goldman and G. Lord, *A new look at entropy and the life table*, Demography **23**, no. 2 (1986), 275–282.
