# Supplementary material for: Measuring the shape of mortality across animals and plants: Alternatives to H entropy metrics reveal hidden type IV survivorship curves and associations with parental care at macro‐ecological scales
Source: Ecol Evol. 2023 May 17;13(5):e10076. doi: 10.1002/ece3.10076 (PMC10191775; doi:10.1002/ece3.10076)
Supplement: Supplementary file 2 — Appendix S2. [file ECE3-13-e10076-s001.docx]

B

A

**Figure S1 Relationship between H-entropy, (a) and AUC, (b) AUC measured using the Rage package for 3,000,000 simulated survivorship curves not corrected for length.** Each graph demonstrates how H-entropy has a concave relationship with AUC (a, b) and in extension with survivorship curve type with low H-entropy values representing both type I and type IV survivorship curves.

**Figure S2**: **Relationship for simulated data between H entropy and the area under the lx curve as a percentage of the highest possible area represented by a square for the most extreme type I curve (AUC) corrected for length of *lx* sequence using 50 evenly spaced inferred points.** Survivorship curves that were generated from randomly generated population matrices (n = 10,000) are highlighted in light grey, data simulated using randomised juvenile and adult mortality are highlighted in dark grey (n = 10,000) while data simulated from the Gompertz model are highlighted in orange (n = 10,000).

**Table S1.** Results of models testing whether the shape of mortality in species with parental care is skewed towards type I survivorship curves as measured using the area under the lx curve, as a proportion of the most extreme type I curve, adjusted for *lx* series length using splines. The estimate (β) is reported as the mode of the posterior distribution with the higher and lower 95% credibility interval (CI). Fixed terms include the intercept and the effect associated with the presence of parental care, with random terms associated with *phylogenetic*, within *species* and residual variation reported as the proportion of each random term.

| **AUC spline model** | Estimate (β) | lower 95% CI | Higher 95% CI |
| --- | --- | --- | --- |
| **Fixed terms** |  |  |  |
| *Intercept* | 0.24 | -0.16 | 0.34 |
| *Parental care present* | 0.08 | 0.01 | 0.15 |
| **Random terms** |  |  |  |
| *Residual* | 0.28 | 0.18 | 0.38 |
| *Species* | 0.56 | 0.35 | 0.73 |
| *Phylogenetic* | 0.01 | 0.00 | 0.40 |

**Table S2.** Results of models testing whether the shape of mortality in species with parental care is skewed towards type I survivorship curves as measured using H-entropy adjusted for *lx* series length using splines. The estimate (β) is reported as the mode of the posterior distribution with the higher and lower 95% credibility interval (CI). Fixed terms include the intercept and the effect associated with the presence of parental care, with random terms associated with *phylogenetic*, within *species* and residual variation reported as the proportion of each random term.

| **H-entropy spline model** | Estimate (β) | lower 95% CI | Higher 95% CI |
| --- | --- | --- | --- |
| **Fixed terms** |  |  |  |
| *Intercept* | 1.15 | 0.39 | 1.77 |
| *Parental care present* | 0.14 | -0.22 | 0.64 |
| **Random terms** |  |  |  |
| *Residual* | 0.11 | 0.06 | 0.25 |
| *Species* | 0.09 | 0.03 | 0.34 |
| *Phylogenetic* | 0.80 | 0.43 | 0.91 |
